# Comparative Analysis of the *Shadoo* Gene between Cattle and Buffalo Reveals Significant Differences

**DOI:** 10.1371/journal.pone.0046601

**Published:** 2012-10-10

**Authors:** Hui Zhao, Lin-Lin Liu, Shou-Hui Du, Si-Qi Wang, Ya-Ping Zhang

**Affiliations:** 1 Laboratory for Conservation and Utilization of Bio-resource, Yunnan University, Kunming, People’s Republic of China; 2 School of Life Science, Yunnan University, Kunming, People’s Republic of China; 3 State Key Laboratory of Genetic Resources and Evolution, Kunming Institute of Zoology, Chinese Academy of Sciences, Kunming, People’s Republic of China; “Mario Negri” Institute for Pharmacological Research, Italy

## Abstract

**Background:**

While prions play a central role in the pathogenesis of transmissible spongiform encephalopathies, the biology of these proteins and the pathophysiology of these diseases remain largely unknown. Since no case of bovine spongiform encephalopathy (BSE) has ever been reported in buffalo despite their phylogenetic proximity to cattle, genetic differences may be driving the different susceptibilities of these two species to BSE. We thus hypothesized that differences in expression of the most recently identified member of the prion family or Shadoo (*SPRN*) gene may relate to these species-specific differences.

**Principal Findings:**

We first analyzed and compared the polymorphisms of the *SPRN* gene (∼4.4 kb), including the putative promoter, coding and 3′ regions, and further verified the entire ORF and putative promoter. This yielded a total of 117 fixed differences, remarkably: 1) a 12-bp insertion/deletion polymorphism in the hydrophobic domain of the cattle but not buffalo gene, introducing a four amino acid expansion/contraction in a series of 5 tandem Ala/Gly-containing repeats; 2) two fixed missense mutations (102Ser→Gly and 119Thr→Ala), and three missense mutations (92Pro>Thr/Met, 122Thr>Ile and 139Arg>Trp) in the coding region presenting different (P<0.05) genotypic and allelic frequency distributions between cattle and buffalo; and, 3) functional luciferase-reporter experiments for the predicted promoter region, consistent with a significantly higher activity in buffalo than cattle. Supporting these findings, immunoblotting revealed higher relative expression levels of Sho protein in cerebrum from buffalo than from cattle. In addition, for cattle, highest Sho expression was detected in obex, as compared to cerebrum or cerebellum.

**Significance:**

Our findings support Sho as a non-PrP specific marker for prion infections, with obex as the best tissue source for the detection of Sho in TSE rapid tests. Moreover, these discoveries may prove advantageous for further understanding the biology of prion diseases.

## Introduction

Transmissible spongiform encephalopathies (TSEs), also known as prion diseases, are a class of fatal neurodegenerative maladies that affect various mammals, including cattle, sheep, mink, cervids, and humans. The epidemics of bovine spongiform encephalopathy (BSE) and of its human form termed variant Creutzfeldt-Jakob disease (vCJD) during the last two decades drew special interest to the study of prion borne diseases. All TSEs are characterized by the conversion of the normal host’s cellular prion protein (PrP^C^) into an abnormal protease-resistant isoform (PrP^Sc^) followed by its pathological accumulation [1]. Present knowledge supports the notion that the host’s PrP^C^ (encoded by prion protein gene, *PRNP*) plays a central role in prion diseases since its expression is absolutely required for disease progression [2], [3]. However, the exact physiological function of PrP^C^ and how it associates with additional factors contributing towards the development of these diseases remain largely unknown.

The mammalian prion protein gene family currently comprises two genes in addition to the above mentioned *PRNP*, that is, prion protein doppel gene (*PRND*; encoding for doppel protein, Dpl) and shadow of prion protein gene (*SPRN*; encoding for shadoo protein, Sho) [4]. The *PRND* gene is expressed almost exclusively in testes and heart [5], and reportedly plays little role in TSE pathology. Conversely, *SPRN,* the most recently identified member of the prion gene family, encoding for a protein (Sho) that is highly conserved among many species, from fish to mammals [4], may be functionally related to the development of prion diseases. In this regard, Sho displays several similarities with prion protein (PrP), including a highly conserved N-terminal signal sequence, a hydrophobic domain (HD) in the middle of the protein, and a C-terminal signal sequence for glycophosphotidylinositol (GPI)-anchor attachment [4]. Sho is predominantly expressed in brain tissue [4], the main target organ in prion diseases. Notably, a positive correlation has been found between *SPRN* and *PRNP* mRNA expression in sheep cerebrum and cerebellum, suggesting a co-regulation of the genes in these tissues [6]. Interestingly, Sho protein levels are profoundly reduced while PrP^Sc^ accumulates in the brains of prion-infected rodents and in infected neuroblastoma cells, suggesting that Sho depletion is an indicator or tracer for a pathogenic event with its expression influencing the pathophysiology of TSEs [7], [8], [9]. The inverse relationship observed between Sho and PrP^Sc^ expression levels may also point to a regulatory cross-talk pathway between the two proteins [10].

Nevertheless, recent studies in a mouse model have not supported the hypothesis of Sho protein playing a role as a prion disease modifier, since: a) the down-regulation of Sho and development of clinical prion disease were not synchronized [8], [9]; b) Sho levels following prion infection in transgenic mice overexpressing mouse Sho Tg(MoSho) were higher than those present in uninfected, wt mice [8], [9]; and, c) knocking out Sho protein did not affect the incubation time of the prion-borne disease [11]. While these studies do not support a direct causative role of Sho depletion in the development of prion disease, at least in a research mouse model, several arguments can still be put forth supporting a relationship with the development of prion disease. First, Sho reduction is a prion disease-specific event because while its levels were reduced in different mammalian species infected with different prion strains, the protein’s levels were unaltered in the brains of transgenic (Tg) mice following accumulation of aggregated proteins typical for other neurodegenerative diseases [8], [9]. Second, analyses of prion-infected mice revealed that down-regulation of Sho protein was not related to *SPRN* mRNA abundance at any stage in prion infection [8], [9], suggesting increased turnover rather than decreased translation as the cause for the decreased protein level. In this regard, differences in the extent of Sho depletion between various prion strains were associated with relative amounts of the PrP^Sc^ C2 proteolytic fragment [8]. Third, co-immunoprecipitation experiment showed that the hydrophobic tract and C-terminal domains of Sho were sufficient for the PrP^Sc^-mediated interactions that result in Sho reduction during prion disease [8]; this may relate to the conformational change observed in PrP^Sc^ during prion disease. And, finally, Sho was regarded as a vCJD suppressor following the description of putative *SPRN* null alleles in two patients suffering from this disease [12]. Thus, while Sho seems not to be a mere bystander during the development of prion-borne diseases [8], the underlying mechanisms for Sho depletion in prion-infected brains and its exact role as it correlates to the development of prion disease remain to be unraveled. Further studies on *SPRN* gene or its protein Sho could strengthen our understanding regarding prion disease and may provide useful in deciphering several unresolved facets of prion biology.

Regarding differences in prion disease-related gene sequence among domestic species, cloning of the bovine and ovine *SPRN* gene revealed a high level of homology [13], [14]. Studies of the *SPRN* gene in *Bos taurus* have included mapping by comparative analysis of the locus and of the predicted flanking genes [13]. However, unlike the ovine, a bovine population has not been screened to identify potential *SPRN* gene polymorphisms.

The domestic buffalo, *Bubalus bubalis*, has been an integral part of livestock agriculture in Asia for over 5000 years for all its pulling power, as well as milk, meat and hide production. Even today, there are 172 million heads of buffalo in the world, of which 22.3 million are in China thus accounting for 13% of the total population (FAO statistics). One remarkable fact is that not a single case of BSE has ever been reported in buffalo, compared to more than 190 thousand cattle infected by BSE up to date worldwide (OIE statistics). Although the main risk factor for BSE in cattle is environmental, i.e. exposure to feedstuffs contaminated with the infectious agent, the genetic component reportedly plays a substantial role in the susceptibility/resistance to prion diseases in both humans and domestic species. For instance, missense polymorphisms in sheep *PRNP* at codons 136, 154 and 171 are strongly correlated with disease susceptibility and progression in animals affected by natural scrapie [15]. Moreover, mutations in the human *PRNP* gene are linked to over 30 inherited forms of human prion diseases [16]. To date, most analyses of bovine populations for specific TSE susceptibility factors have focused on breeds derived from BSE-susceptible cattle. Only two reports have shown that Anatolian and Pakistani buffalo breeds displayed significant differences in *PRNP* indel polymorphisms associated with disease susceptibility as compared to cattle [17], [18]. However, no study has addressed sequence analyses of the *SPRN* gene in buffalo. Therefore, this study was designed to investigate whether a genetic component was associated with the different resistance/susceptibility to prion disease observed between cattle and buffalo. We analyzed the 5′ region, exon 1, intron, open reading frame (ORF) and 3′ region of the *SPRN* gene by contrasting these findings between the two species. Our data unraveled a few but apparently critical, fixed and significant differences between the cattle and buffalo *SPRN* genes. These findings may prove useful in understanding the biology of prion disease as it possibly relates to species-specific susceptibility.

## Results

### PCR and Sequencing

Primers used for PCR and sequencing in cattle, were designed based upon the *SPRN* sequence of *Bos taurus* (GenBank accession No. DQ058606). For sequencing the buffalo homologue, we first compared the *SPRN* sequences between cattle and sheep (GenBank accession No. DQ870545), and selected the conserved regions to design suitable primers. Once the buffalo *SPRN* gene was cloned, specific primers could be designed for further sequencing analysis.

Reportedly, gene density and GC content are much higher in the *SPRN* genomic environment than in other genes attached to the prion protein gene family [19]. Unfortunately, PCR-amplification and sequencing of GC-rich templates are often hampered by the formation of stable secondary structures like hairpins. In this study, the addition of DMSO to the reaction solution did not help. In an attempt to solve these problems, we first obtained the overlapping amplicons of *SPRNA* (2317 bp) and *SPRNB* (2862 bp) (Figure S1 and Table S1) using the GC-RICH PCR System. Then, with these amplicons as template, sequencing was performed using the primers listed in Table S2. Nevertheless, the extreme GC-content of the regions around the predicted promoter and intron made sequencing difficult. Therefore, we amplified four small segments, namely *SPRNA*-b, -c, -d, and -g (Figure S1 and Table S1), using genomic DNA as template, and then cloned them into a T-vector for sequencing. The resulting ∼4.4 kb fragments corresponding to the *SPRN* gene were sequenced in 17 cattle and 11 buffaloes. Our results revealed the *SPRN* gene structure, size and GC-content to be similar between buffalo and cattle (Table 1). In addition, the average GC-content of the bovine *SPRN* genes (69%) was much higher than that in mouse and human (58% and 66%, respectively). Specifically, GC content was particularly high in the exon 1 (81%) region of the bovine *SPRN* genes.

**Table 1:** Summary of the *SPRN* gene structure and GC-content in cattle, buffalo, human and mouse.

	Total		Exon 1		Intron		Exon 2		CDS	
	GC (%)	Size (bp)	GC	Size	GC	Size	GC	Size	GC	Size
Cattle	69	4430 *	81	111	73	726	76	599	77	432
Buffalo	69	4454 *	81	111	73	725	77	599	78	432
Human	66	3913	78	101	71	779	64	3033	79	456
Mouse	58	2203	62	148	57	876	58	1178	68	444

* Indicates the shortest size sequenced among 17 cattle or 11 buffaloes in this study.

The human and mouse *SPRN* gene sequences were obtained from GenBank accession no. BN000518 and BN000519, respectively.

CDS  =  coding sequence; GC  =  Guanine and Cytosine.

### Mutation Screening

Sequence analysis of the *SPRN* gene cloned from 17 healthy cattle and 11 healthy buffaloes, revealed 119 fixed differences between the two species. Except for those in the 3′ region, all other fixed differences between the two homologues are listed in Table 2. For convention, nucleotide numbers listed herein and throughout this manuscript refer to the *SPRN* sequence of *Bos taurus* (GenBank accession No. DQ058606). Overall, fifteen, three and seventeen mutations were distributed along the 5′, exon 1 and intron regions, respectively. Additionally, nine mutations were detected in the coding sequence (CDS) that we denote with “→” or “>” symbols in order to differentiate between inter-species fixed mutations and intra- and/or inter-species polymorphisms, respectively. Thus from these mutations, three were nonsynonymous (92 Pro→Thr, 102Ser→Gly and 119Thr→Ala), while six were synonymous (6T→C, 75G→A, 120C→T, 177G→T, 285A→G and 319A→C; number relative to the ORF nucleotide) substitutions. Moreover, 75 fixed differences were found between cattle and buffalo within the 3′ region of the *SPRN* gene (Table S3).

**Table 2:** Overview of the fixed differences in the 5′ region, exon 1, intron and open reading frame (ORF) of *SPRN* gene between cattle and buffalo.

Mutation*	Cattle	Buffalo	Location	Amino acidchange
g.967	A	G	5′ region	
g.986	C	T		
g.987	T	C		
g.997	C	G		
g.1045	G	A		
g.1066	C	A		
g.1075	A	G		
g.1088	G	C		
g.1093	G	del		
g.1096	A	G		
g.1100	G	A		
g.1143	G	T		
g.1161	A	C		
g.1196	C	T		
g.1202	T	G		
g.1388	A	G	Exon 1	
g.1452	G	C		
g.1458	C	T		
g.1500	C	A	Intron	
g.1598	C	G		
g.1638	G	A		
g.1664	G	del		
g.1672	A	G		
g.1768	G	A		
g.1799	C	T		
g.1800	A	G		
g.1814	G	C		
g.1836	G	A		
g.1856	T	A		
g.1878	T	C		
g.1935	C	T		
g.1976	A	C		
g.2004	G	A		
g.2102	G	A		
g.2171	A	G		
g.2213	T	C	ORF	
g.2282	G	A		
g.2327	C	T		
g.2384	G	T		
g.2481	C	A		Pro92Thr
g.2492	A	G		
g.2511	A	G		Ser102Gly
g.2526	A	C		
g.2562	A	G		Thr119Ala

* Nucleotide number corresponds to the *SPRN* sequence of *Bos Taurus* (GenBank accession No. DQ058606.

Within species, we detected a total of 112 polymorphisms (Table S4) from which 2 were present in both cattle and buffalo, 66 were buffalo-specific and 44 were cattle-specific. Sequence analysis of the 5′ region yielded nine mutations from which 6 were buffalo-specific and 3 were cattle-specific. Notably, four buffalo-specific single nucleotide polymorphisms (SNPs; g.1077G>C, g.1079C>A, g.1244C>A and g.1245T>C) and one cattle-specific SNP (g.1164C>A) showed significant differences in both genotypic and allelic frequency distributions. In addition, there were 2 mutations in exon 1 both of which were rare SNPs and present only within cattle breeds. Intron 1 presented seventeen mutations with most of them being buffalo-specific variants. Worth noting, however, were the mutations detected in the CDS. For instance, two mutations caused an amino acid change (122Thr>Ile and 139Arg>Trp) in the predicted amino acid sequence of Sho in both cattle and buffalo, and in buffalo, respectively. Importantly, a 12-bp insertion polymorphism affecting the coding region of *SPRN* was found in cattle only. This mutation resulted in a four amino acid expansion in a series of 5 tandem Ala/Gly-containing repeats affecting the structure of the hydrophobic domain (HD). Lastly, analysis of the 3′ region yielded 77 SNPs, of which 44 were buffalo-specific variants.

### Mutations Spanning the Coding Region

To determine whether additional SNPs differed between the two species, as well as to assess whether the buffalo gene displayed the 12-bp insertion polymorphism detected in cattle, the entire *SPRN* coding region was further sequenced in 202 cattle and 167 buffaloes. This yielded 11 additional SNPs not identified during the initial screening; notably, seven of these resulted in a predicted amino acid change.

Therefore, a total of 26 SNPs and a 12-bp insertion/deletion (indel) polymorphism were detected in the coding region of the gene of interest among the cattle and buffalo samples analyzed. Regarding SNPs, half of them were synonymous substitutions (Table 3). In addition, six silent mutations (6T→C, 75G→A, 120C→T, 177G→T, 285A→G and 319A→C) detected during the initial screening were further verified to be the fixed differences between cattle and buffalo after enlarging the sample size. Moreover, 3 silent substitutions (96T>C, 228T>A and 357G>A) were only detected within the buffalo species, whereas 4 silent substitutions (189G>T, 201C>G, 288A>G and 360G>A) were cattle-specific mutations. Conversely, 13 mutations resulted in a predicted amino acid change (Table 4) with two amino acid changes (102Ser→Gly and 119Thr→Ala) detected during the initial screening confirmed to be fixed differences between the two species during this round of sequencing. Furthermore, two missense mutations (122Thr>Ile and 139Arg>Trp) differed significantly in genotypic and allelic frequency distributions between cattle and buffalo. In addition, residue 92 which was a proline in cattle, corresponded to two different amino acids in buffalo, namely threonine (85.65%) or methionine (14.35%). Notably, there were six buffalo-specific missense mutations (79Ser>Trp, 90Leu>Pro, 92Pro>Thr/Met, 119Thr>Ala, 123Gly>Ser and 139Arg>Trp), indicating that most of nonsynonymous substitutions were located within the C-terminus of Sho in this species. Conversely, four rare missense mutations (63Ala>Val, 87Pro>Thr, 88Ala>Pro and 142Arg>Gln) were detected within this region of the cattle gene.

**Table 3:** Comparison of genotype frequencies of silent mutations in the open reading frame of *SPRN* gene in cattle and buffalo.

			Frequency			Frequency			Frequency	
Position#	Amino acid change	Genotype	Cattle	Buffalo	Genotype	Cattle	Buffalo	Genotype	Cattle	Buffalo
6	***Asn2Asn***	T/T	1.000	0.000	C/C	0.000	1.000			
75	***Lys25Lys***	G/G	1.000	0.000	A/A	0.000	1.000			
96	*Arg32Arg	T/T	1.000	0.820	T/C	0.000	0.144	C/C	0.000	0.036
120	***Gly40Gly***	C/C	1.000	0.000	T/T	0.000	1.000			
177	***Val59Val***	G/G	1.000	0.000	T/T	0.000	1.000			
189	Ala63Val	G/G	0.995	1.000	G/G	0.050	0.000			
201	Ala67Ala	C/C	0.955	1.000	C/G	0.045	0.000			
228	Ala76Ala	T/T	1.000	0.994	T/A	0.000	0.006			
285	***Ala95Ala***	A/A	1.000	0.000	G/G	0.000	1.000			
288	*Glu96Glu	A/A	0.183	0.000	A/G	0.248	0.000	G/G	0.509	1.000
319	***Arg107Arg***	A/A	1.000	0.000	C/C	0.000	1.000			
357	Thr119Thr	G/G	1.000	0.168	G/A	0.000	0.407	A/A	0.000	0.425
360	Gly120Gly	G/G	0.991	1.000	G/A	0.005	0.000	A/A	0.005	0.000

Letters in boldface and italics denote fixed differences between cattle and buffalo.

# Nucleotide number corresponds to the coding region of *SPRN* sequence.

* Denotes significant differences in genotype frequencies between cattle and buffalo.

**Table 4:** Comparison of the genotype frequencies of missense mutations and indel polymorphisms in the open reading frame of *SPRN* gene between cattle and buffalo.

			Frequency	
Position#	Amino acidchange	Genotype	Cattle	Buffalo
188	Ala63Val	C/C	0.995	1.000
		C/T	0.005	0.000
199	indel AAAG	wt/wt	0.941	1.000
		12-bp ins/12-bp ins	0.005	0.000
		12-bp del/wt	0.010	0.000
		12-bp ins/wt	0.045	0.000
236	Ser79Trp	C/C	1.000	0.988
		C/G	0.000	0.012
259	Pro87Thr	C/C	0.995	1.000
		C/A	0.005	0.000
262	Ala88Pro	G/G	0.995	1.000
		G/C	0.005	0.000
269	Leu90Pro	T/T	1.000	0.988
		T/C	0.000	0.012
274 275	*Pro92Thr/Met	C/C C/C	1.000	0.000
		A/A C/C	0.000	0.725
		A/A C/T	0.000	0.263
		A/A T/T	1.000	0.012
***304***	***Ser102Gly***	A/A	1.000	0.000
		G/G	0.000	1.000
***355***	***Thr119Ala***	A/A	1.000	0.000
		A/G	0.000	1.000
		G/G	0.000	1.000
365	*Thr122Ile	C/C	0.985	0.162
		C/T	0.015	0.413
		T/T	0.000	0.425
367	Gly123Ser	G/G	1.000	0.922
		G/A	0.000	0.066
		A/A	0.000	0.012
415	*Arg139Trp	C/C	1.000	0.689
		C/T	0.000	0.257
		T/T	0.000	0.054
425	Arg142Gln	G/G	0.965	1.000
		G/A	0.035	0.000

# Nucleotide number corresponds to the coding region of *SPRN* sequence.

* Denotes significant differences in genotype frequencies between cattle and buffalo.

Letters in boldface and italics indicate fixed differences between cattle and buffalo.

Table 5 shows how nucleotide sequences of each repeat were compared in the HD of the *SPRN* gene. Different repeats were recognized by their distinct nucleotide sequences. Therefore, although R2, R3 and R4 displayed identical amino acid sequences, the corresponding nucleotides were variants of the repeat sequence. Notably, the insertion or deletion of 12 bp caused the contiguous repeat or deletion of the R3 sequence repeat (gcC gcG gcg ggg; Table 5), respectively. Thus, the predicted sequence repeat orders for the 12-bp indel polymorphism in bovine Sho were represented as follows: a) Cattle/buffalo wt: R1-R2-R3-R4-R5; b) Cattle −12 nt: R1-R2-R4-R5; and, c) Cattle +12 nt: R1-R2-R3-R3-R4-R5. Moreover, this 12-bp indel polymorphism was only found within cattle, but not buffalo breeds. The genotypic frequency of the 12-bp indel polymorphism for the bovine *SPRN* gene is shown in Table 4. Remarkably, the sequence with five repeats was most prevalent in cattle, with mean genotypic and allelic frequencies of 0.941 and 0.968, respectively. Conversely, mean genotypic (homozygous) and allelic insertion frequencies were 0.045 and 0.027, respectively. Interestingly, we did not find a homozygous genotypic deletion, but the deletion/wt heterozygous genotypic and allelic deletion frequencies were 0.010 and 0.005, respectively. Worth noting is the significant difference in the genotypic frequency distribution (p = 0.016) and the marginal difference in the allelic frequency distribution (p = 0.053) detected for the 12-bp indel polymorphism between cattle and buffalo.

**Table 5:** Comparison of the repeat nucleotide sequences in the hydrophobic domain of bovine *SPRN* gene.

Species	Repeat #	Amino acid sequence	Nucleotide sequence
Cattle/buffalo wt	R1	VAAG	gTg gcc gcg ggg
Cattle/buffalo wt	R2	AAAG	gcg gcG gcg ggg
Cattle/buffalo wt	R3	AAAG	gcC gcG gcg ggg
Cattle/buffalo wt	R4	AAAG	gcA gcc gcA ggC
Cattle/buffalo wt	R5	LAAG	CTg gcT gcg ggC

wt  =  wild type.

### *In silico* analysis

The buffalo *SPRN* gene sequence was initially generated to contain inter-specific fixed mutations, but no intra-specific polymorphic changes. Next, we performed *in silico* analysis of the corresponding buffalo and cattle (GenBank accession No. DQ058606) sequences using TFSEARCH and Promoter Scan programs. Interestingly, while this analysis revealed that the predicted promoters of cattle and buffalo had no TATA- or CCAAT-box, a large number of putative transcription factor binding sites such as specificity protein 1 (Sp1), activator protein 1 (AP-1), and activator 2 (AP-2) were identified. Taking into account the fixed differences between cattle and buffalo, we found 20 differences in putative transcription factor binding sites around the predicted promoter and intron regions (Table 6). Therefore, as a result of five mutations, we predicted that the buffalo *SPRN* gene would lose six binding sites for core binding factor α, variant 1a (AML-1a), P300, AML-1a and CP2, Ikaros transcript 2 (Ik-2) and Sp1 transcription factors, respectively. Apart from Sp1, all other transcription factors show specific expression in tissues other than brain, where Sho is highly expressed [13]. For instance, the AML-1a factor targets a sequence present in a number of viral enhancers as well as T-cell-specific promoters and enhancers [20]. By contrast, the buffalo *SPRN* gene would gain 14 binding sites due to nine specific mutations (Table 6). Intriguingly, three of the corresponding transcription factors, namely AP-2, USF and Sp1, are ubiquitous and activate a wide range of viral and cellular genes [21]–[23]. In addition, GATA-3, which targets sites in promoters and/or enhancers [24], as well as an unknown factor associated with the JCV_repeated_sequence [25], have also been found to be highly expressed in brain. We hypothesize that these buffalo-specific transcription factor binding sites may be increasing the expression of *SPRN* in buffalo brain tissue.

**Table 6:** Comparison of putative transcription factor bind sites in *SPRN* gene between cattle and buffalo.

Transcription factor	Binding site position*	Mutation†	Strand	Binding site	Binding site in cattle	Binding site in buffalo
AML-1a	−368	g.987T>C	+	TGCGGT	have	no
GATA-3	−293	g.1066C>A	+	GGGATTGGG	no	yes
CP2	−287	g.1075A>G	−	TGGGGTCGGGC	no	yes
P300	−214	g.1143	+	GCGGGGGGTGCGA	yes	no
JCV_repeat_sequence	−157	g.1202T>G	+	GGGTGGGG	no	yes
PuF	−157	g.1202T>G	+	GGGTGGG	no	yes
AP-2	−157	g.1202T>G	−	GGGTGGGG	no	yes
AML-1a	−153	g.1202T>G	+	TGGGG	yes	no
CP2	−153	g.1202T>G	-	T GGGGGTGCGC	yes	no
Ik-2	+309	g.1638G>A	+	GGGTGGGACT	no	yes
Ik-2	+309	g.1672A>G	+	GCCTGGGAAAG	yes	no
USF	+438	g.1799–1800CA>TG	+	CCACATG	no	yes
USF	+441	g.1799–1800CA>TG	−	CCACATG	no	yes
MZF1	+456	g.1814G>C	−	CCCCAGG	no	yes
GATA-1, GATA-2	+496	g.1856T>A	−	CCCATCCTC	no	yes
Sp1	+496	g.1878T>C	−	CCACGCCCC	no	yes
Sp1	+645	g.2004G>A	−	GCCCGCCCC	yes	no
RREB-1	+638	g.2004G>A	+	CCCCAGCCCACCC	no	yes
AML-1a	+646	g.2004G>A	−	ACCCCA	no	yes

* The binding site position is given with respect to the transcription factor start site (position 1355 in GenBank accession number DQ058606).

† Mutation indicates the nucleotide change from the cattle to the buffalo sequence. For example, g.987T>C indicates that in position 987 there is a T and C in the sequences of cattle and buffalo, respectively.

### Functional Analysis

**Figure 1:**
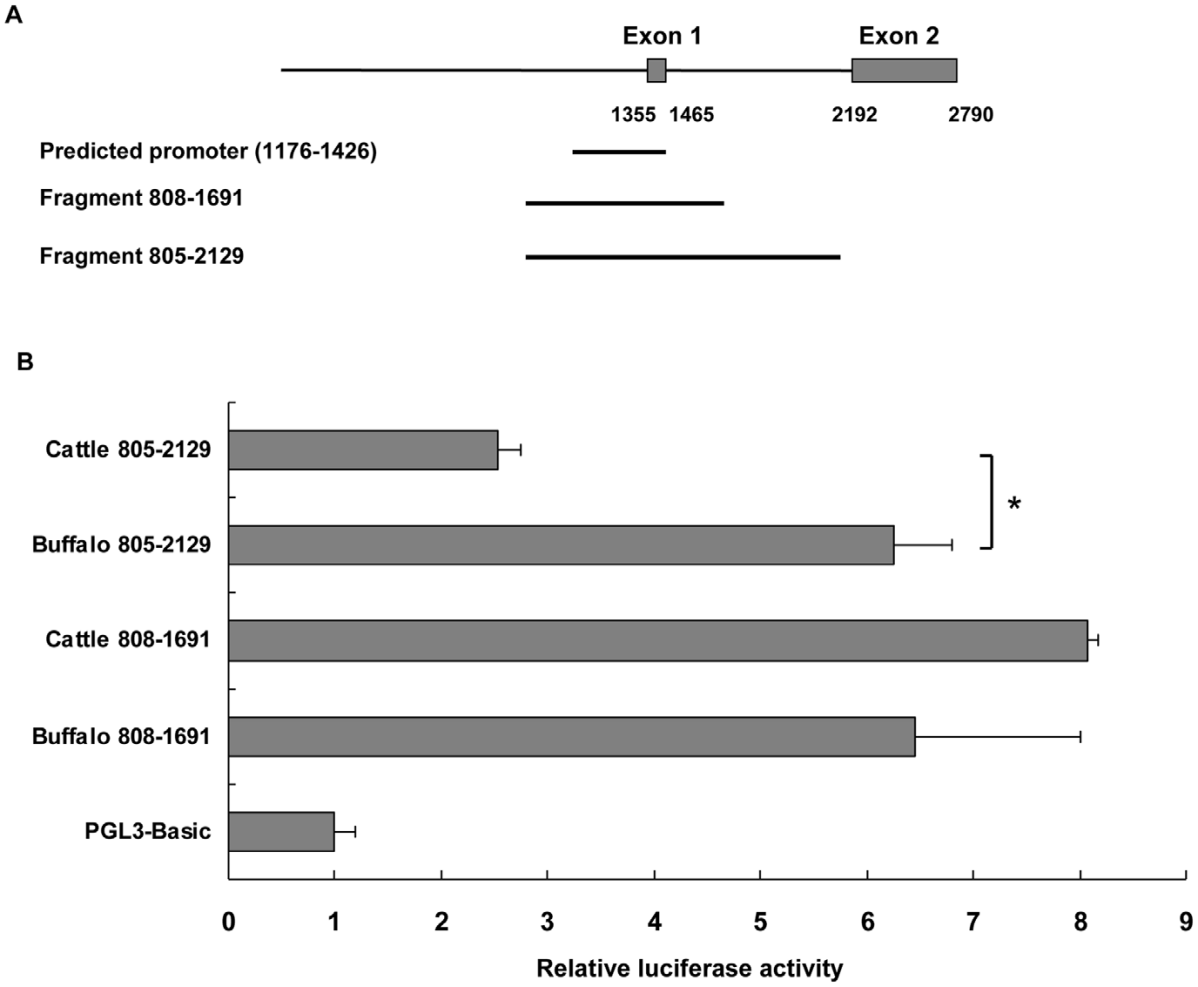
Relative luciferase activity of the constructs containing the putative *SPRN* promoter. (A) Schematic representation of the bovine *SPRN* fragments used in this study. The promoter activity of two constructs (containing fragments g.808–1691 and g.805–2129, respectively) was assessed. The two boxes are indicated as exon 1 and 2 of the bovine *SPRN* gene spanning from g.1355 to 1465 and from g.2192 to 2790, respectively. (B) Relative luciferase activity of the cattle or buffalo *SPRN* promoter in N2a cells. The data show the constructed plasmid activity after normalization with the co-transfected reference vector (pRL-TK), and relative to the activity of pGL3-Basic vector, which the activity was set to 1. Results are shown as mean ± SEM of three independent experiments. Each independent experiment was performed with three separately transfected wells. * Indicates significant differences for relative luciferase activity between the constructs (g.805–2129) in cattle and buffalo.

To compare the promoter activity between the cattle and buffalo genes, luciferase reporter plasmids were constructed and transfected into N2a cells. Using the Promoter Scan program, the forward strand spanning g.1176 to g.1426 was predicted as the putative promoter region for both the cattle and buffalo sequences. Conversely, Lampo et al. had suggested the ovine *SPRN* promoter to be located in a more 5′ distant region, spanning g.739–1259 [26] (similar to bovine *SPRN* g.845–1407). Based upon this information, we first constructed the pGL3Basic reporter plasmid containing the fragment g.808–1691 to cover both possibilities. Moreover, *in silico* analysis had revealed several differences in putative transcription factor binding sites between the cattle and buffalo *SPRN* gene, ranging from positions g.1799 to 2004 (Table 6). Therefore, we also constructed a reporter plasmid containing fragments g.805–2129 and including the predicted promoter region, exon 1 and intron 1 of the bovine *SPRN* sequence (Fig. 1A). Results were consistent with fragment g.808–1691 driving transcription of the firefly luciferase reporter more efficiently than the parental pGL3Basic plasmid (Fig. 1B). In particular, the plasmid containing the cattle fragment g.808–1691 yielded a reporter output about 8-fold higher than the control plasmid. However, the plasmid containing the large putative cattle promoter fragment g.805–2129 displayed markedly decreased luciferase expression as compared to the plasmid containing fragment g. 808–1691. This finding suggested that some undiscovered transcription factor binding sites located in the region of g.1692–2129 may be suppressing the transcription of the luciferase reporter. Interestingly, plasmids containing the same two fragments of the *SPRN* buffalo gene did not display different luciferase reporter activities. Moreover, when comparing the plasmids containing the large putative promoter fragment (g.805–2129), the buffalo displayed significantly higher luciferase activity than the cattle homologue. Based upon the above findings, we hypothesized that the region spanning g.1692–2129 may contain suppressor-like regions in cattle, while species-specific putative transcription factor binding sites could enhance the promoter activity in buffalo (Table 6). For instance, Sp1 and USF which are ubiquitous proteins and are involved in the regulation of many promoters [21], [22] could be potential candidates driving the activity in the corresponding fragments of the buffalo constructs.

In order to ascertain whether sequence variations in the promoter region between cattle and buffalo were fixed differences, the *SPRNA*-d (g.1458–1883) and *SPRNA*-e (g.1711–2155) fragments (Fig. S1), spanning the putative buffalo-specific transcription factor binding sites, were further sequenced in 115 cattle and 126 buffaloes. Results confirmed that the mutations detected during the initial screening (Table 2) were indeed fixed differences between the two species; that is, with the exception of two SNPs (g.1664 G>del/G and 1856 T>A/R), which were buffalo-specific polymorphisms. The g.1664G>del mutation had no influence on the putative transcription factor binding sites; conversely, the g.1856T>A mutation would confer a binding site for transcription factor of GATA-1 or GATA-2 (Table 6). Interestingly, in 137 buffaloes (plus the initial 11 buffaloes) we did not find a homozygous genotype G/G in g.1856, and the A/A homozygous and A/G heterozygous genotypic frequencies were 0.920 and 0.080, respectively.

**Figure 2:**
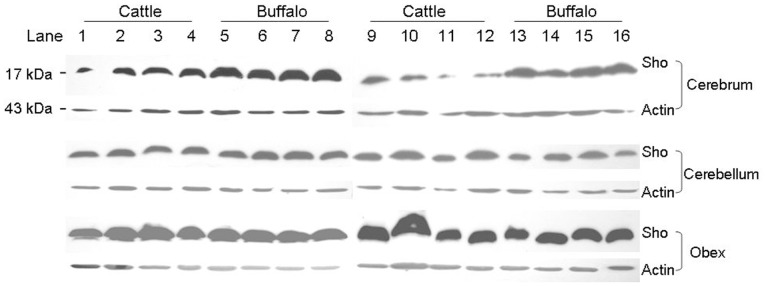
Sho protein expression levels in cerebrum, cerebellum and obex tissues from cattle and buffalo. Homogenates of cerebrum, cerebellum and obex from each 8 cattle and buffaloes were subjected to electrophoresis using 12% polyacrylamide gels and immunoblotted for Sho protein. In order to compare the expression differences between cattle and buffaloes, samples were run simultaneously in groups of 4 for a total of 12 replicates. Immunoblotting for Sho protein yielded a protein of ∼17 kDa, which is consistent with the detection of Sho. Lanes 1–4 and lanes 9–12 correspond to cattle samples; lanes 5–8 and lanes 13–16 correspond to buffalo samples. Actin was blotted as a loading control. The relative expression levels of Sho protein were similar in cerebellum and obex between cattle and buffalo, but higher in the cerebrum of buffalo as compared to cattle.

Next, to investigate potential differences in relative expression levels of Sho protein between cattle and buffalo, three central nervous system (CNS) tissues including cerebrum, cerebellum and obex were submitted to immunoblotting. Because no anti-Sho antibody raised against cattle is commercially available, herein we used an antibody raised against the epitope corresponding to amino acids 83–113 on the C-terminal region of human Sho. As shown (Fig. 2), the proteins were effectively recognized by this antibody, given the highly conserved nature of this epitope between the two species. Moreover, in cattle, highest Sho protein expression was identified in the obex region. Interestingly, while Sho expression was similar for cerebellum and obex between cattle and buffalo, in cerebrum expression levels were higher in buffalo (Fig. 2).

## Discussion

Transmissible spongiform encephalopathies (TSEs) comprise a group of fatal diseases affecting multiple species that include scrapie in sheep and goats, chronic wasting disease in mules, deer and elk, BSE in cattle, as well as Kuru and CJD in humans. Since buffalo are ruminants belonging to the same phylogenetic family as cattle, there is no apparent reason to envision an exempted susceptibility to BSE. However, despite large numbers of buffalo heads in production worldwide paired with a longer lifespan as compared to cattle and hence more opportunity for prion replication, there has been no single case of BSE ever recorded in buffalo. For example, Italy has a 120,000 head-count and no history of BSE in their buffalo population, while 48 cases were identified in cattle during 2001 (OIE statistics). These facts are of paramount importance for understanding the pathophysiology of TSEs and raise the question of whether genetic differences between cattle and buffalo could account for the different susceptibilities to developing BSE. In this regard, studies in cattle comparing genetic data from BSE-diseased and healthy animals have shown that a 23-bp indel in the *PRNP* promoter [27], [28] and a 12-bp indel in intron 1 [29] were associated with disease susceptibility. Further reporter gene assays have demonstrated lower expression levels of the ins/ins allele compared to the del/del allele [30]. Interestingly, most of Anatolian water and Pakistani buffalo (∼ 90%) were shown to have the 23-bp insertion/12-bp insertion (I_23_–I_12_) haplotype, in agreement to our preliminary findings in Chinese buffalo breeds (unpublished data). The higher I_23_–I_12_ haplotype frequency in buffalo, as compared to cattle worldwide, may be responsible for the resistance to BSE observed in buffalo-type breeds.

Indeed, the paradigm of species-specific susceptibility to TSEs has also been well-proven in relation to the canine PrP^C^ (cPrP^C^). While several cases of feline spongiform encephalopathy were reported during the BSE crisis in the United Kingdom [31] and in other countries, there are no reports of TSE-infected dogs. Furthermore, canine-derived Madin-Darby kidney cells show *in vitro* resistance to prion infection [32]. In fact, this apparent resistance of the canine species to prion infection may result from unique structural features in cPrP^C^. In particular, the presence of Asp-159 and Arg-177 in the C-terminal globular domains of the dog protein may cause unique charge distribution patterns that were hypothesized to correlate with the protection against BSE challenge observed in this species [33].

Important structural similarities between Sho and PrP^C^ including N-terminal repeats, a HD, endoproteolysis to a stable C1 fragment, and a C-terminal glycosylation site prefacing a GPI anchor-attachment, suggest that PrP^C^ is an important model for deciphering the structure-to-function characteristics of Sho as it relates to the pathogenesis of prion disease in bovids [10]. To the best of our knowledge, this is the first population screening of the bovine *SPRN* gene including analysis of the putative promoter region, exon 1, intron, ORF and 3′ region. Hence a comparison of the *SPRN* gene (∼4.4 kb) sequence between cattle and buffalo yielded a total of 117 fixed differences. We hypothesize that three important findings derived from our study may hold the key to considering the different susceptibility to BSE encountered between these two species, namely: 1) A 12-bp indel polymorphism in the HD of Sho was identified in cattle but never in buffalo; 2) two amino acid changes (102Ser→Gly and 119Thr→Ala) in the coding region of *SPRN* were found to be fixed in buffalo breeds; and, 3) fixed differences distributed in the predicted promoter and intron 1 regions affecting putative transcription factor binding sites resulted in different species-specific Sho expression levels in cerebrum.

Indeed, the identification of a cattle-specific 12-bp indel polymorphism in the HD of Sho raises the question of whether this could bear relationship with the different susceptibilities of cattle and buffalo to prion infection. Interestingly, both the HD region as well as tandem repeats with positively charged residues within PrP^C^ have been postulated to play a role in the pathogenesis of TSEs. Although the HD is not essential for interaction with PrP^Sc^, it is believed to undergo significant structural changes following prion infection, as antibodies directed toward PrP can detect PrP^C^ but not PrP^Sc^ [34]. Moreover, mutagenesis studies directed towards this highly conserved HD have shown that minor alterations drastically affect the ability of cells to uptake and replicate a prion infection, in both cell culture and transgenic mice models [35]. Similarly, the A117V variant of the palindrome AGAAVAGA in the HD of PrP is linked to Gerstmann-Sträussler syndrome (GSS), a genetic form of human prion disease [36]. Altogether, these studies support the hypothesis that the HD of PrP may play a role in modulating prion toxicity and infectivity [35].

Thus, as reported for PrP^C^ [35], the HD region of Sho contains a series of glycine residues that are highly conserved among all species, strongly suggesting a functional significance. Furthermore, these glycine residues form repeats of two glycines separated by any three residues (i.e. GXXXG) yielding motifs involved in protein-protein interactions in a variety of proteins [35]. In this context, mutations in these glycine-rich regions may result in protein folding derangements linked to central nervous system disorders, such as when amyloid precursor protein gives rise to the amyloid β peptide involved in the pathogenesis of Alzheimer’s disease [37], [38]. Interestingly, mutations involving an alanine expansion in this region of such proteins also resulted in an increased tendency to form aggregates as compared to their wild type counterparts [39], [40]. Moreover, an interaction between Sho’s HD and PrP^C^ residues 108–126 was identified, and thus supported a potential role for Sho’s HD in the physiological function of PrP^C^ and prion pathogenesis [41]. In agreement, a recent study showed that wild type Sho could combine with PrPC to protect cells against physiological stressors and that a mutant devoid of the HD (ShoΔHD) lost this stress-protective activity [42]. Given the physiological importance of the HD of Sho, it is important to note that polymorphisms of this protein’s region have been reported in several species. For instance, three indel variants (−12 nt, +6 nt and +9 nt) in addition to the wild type sequence exist in the HD region of Sho sequenced from several Canadian and American sheep breeds [43]. Similarly, a 12-bp deletion and a 12-bp insertion have been reported in different positions of the HD region of the human *SPRN* gene [12]. While a 12-bp deletion has been described in cattle [44] and ratified in our study (two del/wt heterozygous cases out of 202 cases), we also showed herein the presence of a 12-bp insertion polymorphism (2.7% of the insertion allele frequency) in Chinese cattle breeds. Most striking, however, is that no 12-bp indel polymorphism was found among buffalo breeds; moreover, we show significant differences in genotype frequency distribution of the 12-bp indel polymorphism between cattle and buffalo. Overall, we suggest that the 12-bp indel polymorphism of the HD may play a role in modulating susceptibility/resistance to TSE or/and infectivity to prion in domestic bovids.

A second important conclusion that can be drawn from our study lies in the fact that fixed mutations in the coding region of the buffalo *SPRN* resulted in two amino acid changes (102Ser→Gly and 119Thr→Ala) (Figure S2). Interestingly, species variations in the amino acid sequence of PrP^C^ have been mainly identified in the C-terminus, a domain that is reportedly important for the development of TSEs [33]. Furthermore, at least 20 different mutations in the C-terminal domain of PrP^C^ are presently known to cause inherited prion disease [16]. Interestingly we identified fixed and significant differences between cattle and buffalo that affected the C-, but not the N-terminal region of Sho (Figure S2). For instance, residue 102 is located between the HD and the N-glycosylation site of bovine Sho, which is similar to residue 142 of the human/mouse PrP^C^. Using transgenic mice expressing chimeric murine-ovine PrP^C^ whereby amino acid residue 142 was changed from asparagine to serine, resulted in a large reduction in Me7 prion-induced conversion [45]. Although both amino acids exhibit an uncharged polar side chain, serine is noticeably smaller than asparagine. Furthermore, serine lacks the terminal carboxyamide group, which in asparagine enables the formation of hydrogen bonds and enhances the intrinsic stability of the molecule [45]. Thus, this cell-free conversion experiment demonstrated that the type of residue in the 142 position was important for the assembly of the prion agent. The two Sho protein mutations identified in this study resulted in the change of a polar (Ser, Thr) to a non-polar (Gly, Ala, respectively) amino acid from the cattle to the buffalo predicted protein sequences. While intriguing, further investigation is required to ascertain if these mutations are relevant for the biological properties of Sho. Furthermore, three missense mutations (92Pro>Thr/Met, 122Thr>Ile and 139Arg>Trp) displayed significantly different genotypic and allelic frequency distributions between cattle and buffalo. Residue 92 is located within the C-terminal domain of Sho, while residues 122 and 139 are located within the C-terminal signal sequence. Although no NMR structural studies are available to date for Sho, circular dichroism analyses indicate that this is a natively unstructured protein [7] with the C-terminal domain of Sho located in an analogous position to the three α-helical and two β-strands found in PrP^C^. Notably, the 122Thr>Ile and 139Arg>Trp mutations change a polar to a nonpolar hydrophobic amino acid, and a strongly basic amino acid to a hydrophobic amino acid, respectively. We infer that these changes may introduce significant hydrophobicity and thus affect the biological properties of Sho. Moreover, these mutations may be candidate sites to further investigate the potential functional overlap of Sho with PrP, and thus its role in determining susceptibility to prion disease. Lastly, while others have reported three silent mutations (37C>T, 288A>G and 360G>A) in the coding region of cattle; herein the first mutation was not present but we did identify 7 variants (2 silent mutations and 5 amino acid changes) in addition to the three mutations among cattle breeds [44].

A third important difference lies on the fixed differences spanning the predicted promoter and intron 1 regions as it would relate to putative transcription factor binding sites and hence influence *SPRN* mRNA expression levels. In this study, functional analysis of Sho was consistent with a higher promoter activity in the buffalo transcript. Moreover, we supported this assumption by analyzing Sho expression levels in cerebrum, cerebellum and obex, three tissues known to contain high expression of *SPRN* and *PRNP* mRNA in sheep [46], [47]. Interestingly, for BSE-susceptible cattle the highest relative level of Sho protein was identified in obex, which is in accordance with the higher *PRNP* mRNA expression levels in obex as compared to other CNS samples reported in sheep [47]. Recent reports support a role for Sho protein as a marker or indicator of prion-borne diseases [8], [9]. Hence, our data would support obex as the best source of material for the analysis of Sho in the detection of prion disease which is in agreement with obex being suitable for the detection of PrP^Sc^ in TSE-rapid tests. Attending to the comparison of Sho expression levels between cattle and buffalo, it was particularly interesting that differences were identified for cerebrum but not for cerebellum or obex. We hypothesize that the presence of cerebrum-specific transcription factor(s) controlling the translation of Sho may be responsible for these findings. Although the actual role of Sho in prion disease pathogenesis remains largely unknown, Sho’s activity was inferred to be germane to the maintenance of neuronal viability in postnatal life [11]. It was also reported that inhibition of a PrP^C^ binding protein, Na^+^/K^+^-ATPase [48], resulted in the rapid development spongiform changes similar to those characterizing prion disease [49]. Thus, future studies may be directed at investigating whether the higher Sho expression levels detected in buffalo cerebrum may contribute to this species’ resistance to neurodegenerative malady.

In summary, we present herein the results of a comparative population genomic analysis for the *SPRN* gene between cattle and buffalo. Notably, species-specific indel polymorphisms, mutations, differences in promoter activity and expression levels in cerebrum may provide a different genetic backbone between these two species that may translate to their different susceptibilities to BSE. While many questions remain unanswered, our results are an important step forward in understanding the biology of prion disease as it possibly relates to species-specific susceptibility.

## Materials and Methods

### Animals and Samples

For the initial mutation screening, a total of 28 blood samples including 17 cattle covering 8 different breeds (Dehong, Diqing, Fujian, Guizhou, Hasake, Kunming, Wenshan and Zhaotong) and 11 buffaloes covering 6 different breeds (Chongqing, Dehong, Fujian, Guangdong, Guangnan and Hainan) were used. The full open reading frame (ORF) of the *SPRN* sequence was then analyzed in a total of 369 samples, including 202 cattle and 167 buffaloes. Cattle were from 11 different breeds (Dali, Dehong, Diqing, Fujian, Guizhou, Hasake, Kunming, Qinghai, Wenshan, Zhaotong and Zhongdian) while buffaloes were derived from 7 breeds (Chongqing, Dehong, Fujian, Guangdong, Guangnan, Guizhou and Hainan). Written informed consent for research purposes, approved by the Ethics and Experimental Animal Committee of Kunming Institute of Zoology, Chinese Academy of Sciences, was obtained for all individuals involved in the study.

### Polymerase Chain Reaction (PCR)

Primers for PCR were designed using Primer Premier software (PREMIER Biosoft International, Palo Alto, CA, USA) and oligo 6.0 software (Molecular Biology Insights, Inc., Cascade, CO, USA) based upon the *SPRN* sequence of *Bos taurus* (GenBank accession No. DQ058606). An overview of the primers used in this study with their characteristics is listed in Table S1. The PCR reaction was carried out in 25 µl using the GC-RICH PCR System kit (Roche, Mannheim, Germany) containing 100 ng DNA, 1 unit GC-RICH Enzyme, 200 µM (each) dNTPs, reaction buffer and resolution solution according to the manufacturer’s recommendations. The amplification was carried out in a PCR machine GeneAmp PCR System 9700 (Applied Biosystems, CA, USA) with the following profile: denaturation step at 95°C for 3 min; then 10 cycles of denaturation at 95°C for 30 s, annealing at the corresponding temperature for 30 s, and extension at 72°C for 45 s/kb; followed by 20 cycles of denaturation at 95°C for 30 s, annealing at the corresponding temperature for 30 s, and extension at 72°C for 45 s/kb plus an elongation of 5 s for each additional cycle; and, a final extension at 72°C for 7 min.

### Sequence Analysis

For the initial mutation screening, the overlapping amplicons of *SPRN* fragment A (*SPRNA*) and *SPRN* fragment B (*SPRNB*, Figure S1) were sequenced using the primers listed in Table S2. The high GC-rich regions including *SPRNA*-b, -c, -d and -g (Figure S1 and Table S1) were cloned into the PMD 18-T vector (TaKaRa, Dalian, China). For analysis of the ORF, amplicons were sequenced using the PCR primers listed in Table S1. When sequences were heterozygous for indels in the coding region, the amplified PCR products were also cloned into the PMD 18-T vector. Five clones containing insertions were sequenced with primers in both forward and reverse direction (T-vector-F and T-vector-R, described in Table S2). The sequence analysis was performed with an ABI PRISM 3700 DNA sequencer with BigDye Terminator v3.1 Cycle Sequencing kit (Applied Biosystems, Carlsbad, CA, USA) following the manufacturer’s instructions. Resulting sequences were inserted into GenBank under accession Nos.: JQ811114 to JQ811138 and JQ811158 to JQ811176 for *SPRN* CDS of buffalo and cattle, respectively; JQ811139 to JQ811157 and JQ811177 to JQ811202 for the *SPRN* gene of buffalo and cattle, respectively; and JX482039 to JX482082 and JX482083 to JX482104 for *SPRN* promoter of buffalo and cattle, respectively.

### Luciferase Plasmid Construction

The PCR was performed to amplify the predicted promoter fragments 808–1688 and 805–2129 of *SPRN* using primer pairs of Promoter 1 and Promoter 2, respectively (Table S1). The amplicons containing XhoI and HindIII digestion sites were inserted into the pGL3-Basic Luciferase Reporter Vector (Promega, Madison, WI, USA) following the manufacturer’s protocol. The constructed pGL3Basic vectors were analyzed by sequencing using the RVprimer3 and GLprimer2 (Table S2) as recommended in the pGL3Basic Luciferase Reporter Vector manual.

### Transfections and Luciferase Assay

Mouse neuroblastoma cells N2a were purchased from the Kunming cell bank (Kunming Institute of Zoology, Chinese Academy of sciences, Kunming, China). The N2a cells were grown in Modified Eagle’s Medium (MEM) containing Earle’s salts, L-glutamine, low glucose and 2 mM L-glutamine supplemented with 10% fetal bovine serum, 1% penicillin and streptomycin (Invitrogen, Beijing, China). About 1.3×10^5^ N2a cells were seeded in 24-well plates and co-transfected using the X-tremeGENE HP DNA transfection reagent (Roche, Mannheinn, Germany) with 500 ng of the firefly luciferase reporter plasmid (the constructed pGL3-Basic plasmid) and 50 ng *Renilla* luciferase reporter pRL-TK (Promega, Madison, WI, USA) as a transfection control. Cell lysates were assayed for firefly and *Renilla* luciferase activity 48 h after transfection using the Dual Luciferase Reporter Assay System (Promega, Madison, WI, USA) and a Fluoroskan Ascent FL (Thermo Scientific, Vantaa, Finland). Relative luciferase activities were defined as the ratio of the firefly luciferase to *Renilla* luciferase mean value of each construct relative to the pGL3-Basic vector. Assays were carried out in triplicate from three different transfection experiments.

### Western Blotting

Ten percent (wt/vol) containing 50 mg of CNS tissue (cerebrum, cerebellum and obex) homogenates from each of 8 cattle and buffalo brains were prepared using radioimmunoprecipitation assay (RIPA) buffer (25 mM Tris•HCl pH 7.6, 150 mM NaCl, 1% NP-40, 1% sodium deoxycholate, 0.1% sodium dodecyl sulfate (SDS), Pierce, IL, USA) with the addition of EDTA-free protease inhibitor Cocktail Tablets (Roche, Mannheim, Germany). Protein concentration in the supernatant was determined by the bicinchoninic acid (BCA) protein assay kit (Thermo, IL, USA). Accordingly, aliquots containing 40 µg of protein were loaded per lane, separated by 12% SDS-polyacrylamide gel electrophoresis (PAGE) and then transferred to PVDF membranes (Millipore, MA, USA). The blots were incubated with anti-C-term SPRN Antibody (1∶200, Abgent, CA, USA) or anti-Actin monoclonal antibody (1∶1000, Cell Signaling Technology, MA, USA) and then, incubated with horseradish peroxidase-conjugated secondary antibody (1∶5000, Santa Cruz Biotechnology Inc. California, USA). Immunoblotting against actin served as an internal loading control. Immunoreactive bands were visualized by using SuperSignal West Pico Chemiluminescent Substrate (Thermo, IL, USA) and exposed to X-ray films.

### Sequencing Analysis Software

The DNASTAR software package (Lasergene® Core Suite, Madison, WI, USA) was used for basic handling and analysis of the nucleotide and protein sequencing data. Promoter elements and putative transcription factor binding sites were identified with the program Promoter Scan (http://www-bimas.cit.nih.gov/molbio/proscan) and TFSEARCH ver. 1.3 (http://www.cbrc.jp/research/db/TFSEARCH.html) using the default threshold score of 85.0.

### Statistical Analysis

Differences in frequency distributions between cattle and buffalo were evaluated by chi-square (χ^2^) analysis using SPSS 11.5 software (SPSS Inc., Chicago, Illinois, USA). Differences were considered significant at a p<0.05.

## Supporting Information

Figure S1 Detection of several amplicons of *SPRN* gene in cattle and buffalo. (A) Schematic representation of the PCR products of the bovine *SPRN* gene. The two exons of the bovine *SPRN* gene are represented with boxes. The coding sequence (CDS) region of *SPRN* (grey) is located on exon 2. * Indicates the buffalo-specific fragment amplified by PCR. (B) DNA samples from cattle (Lanes 1–7) and buffalo (Lanes 8–14) were amplified by PCR using the GC-RICH PCR System kit. The extracting PCR products of *SPRNA* (Lanes 1 and 8), *SPRNB* (Lanes 2 and 9) and *SPRN*-cds (Lanes 7 and 14) were directly sequenced. The high GC-rich fragments of *SPRNA*-b (Lanes 3 and 10), *SPRNA*-c (Lanes 4 and 11), *SPRNA*-d (Lanes 5 and 12) and *SPRNA*-g (Lanes 6 and 13) were cloned into the PMD 18-T vector and then sequenced. M: Molecular weight marker.
(TIF)

Figure S2 The predicted amino acid sequences of Shadoo in cattle and buffalo. The amino acid sequence of cattle Sho was translated from the *SPRN* gene sequence DQ058606 of GenBank. The amino acid sequence of buffalo Sho was produced according to the results of population analysis showing fixed differences (underlined) and significant differences (*) between the two species. Dashed box frame denotes the hydrophobic domain of Sho.
(TIF)

Table S1 Amplicon characteristics of the PCR primers used in this study.
(PDF)

Table S2 Primers used for sequencing *SPRN* gene in cattle and buffalo.
(PDF)

Table S3 Overview of the fixed differences in the 3′ region of *SPRN* gene between cattle and buffalo.
(PDF)

Table S4 Distributions of genotype frequency of polymorphisms in cattle and buffalo breeds.
(PDF)
