# Supplementary figures and images for: Comparative Analysis of the Shadoo Gene between Cattle and Buffalo Reveals Significant Differences
Source: PLoS One. 2012 Oct 10;7(10):e46601. doi: 10.1371/journal.pone.0046601 (PMC3468620; doi:10.1371/journal.pone.0046601)

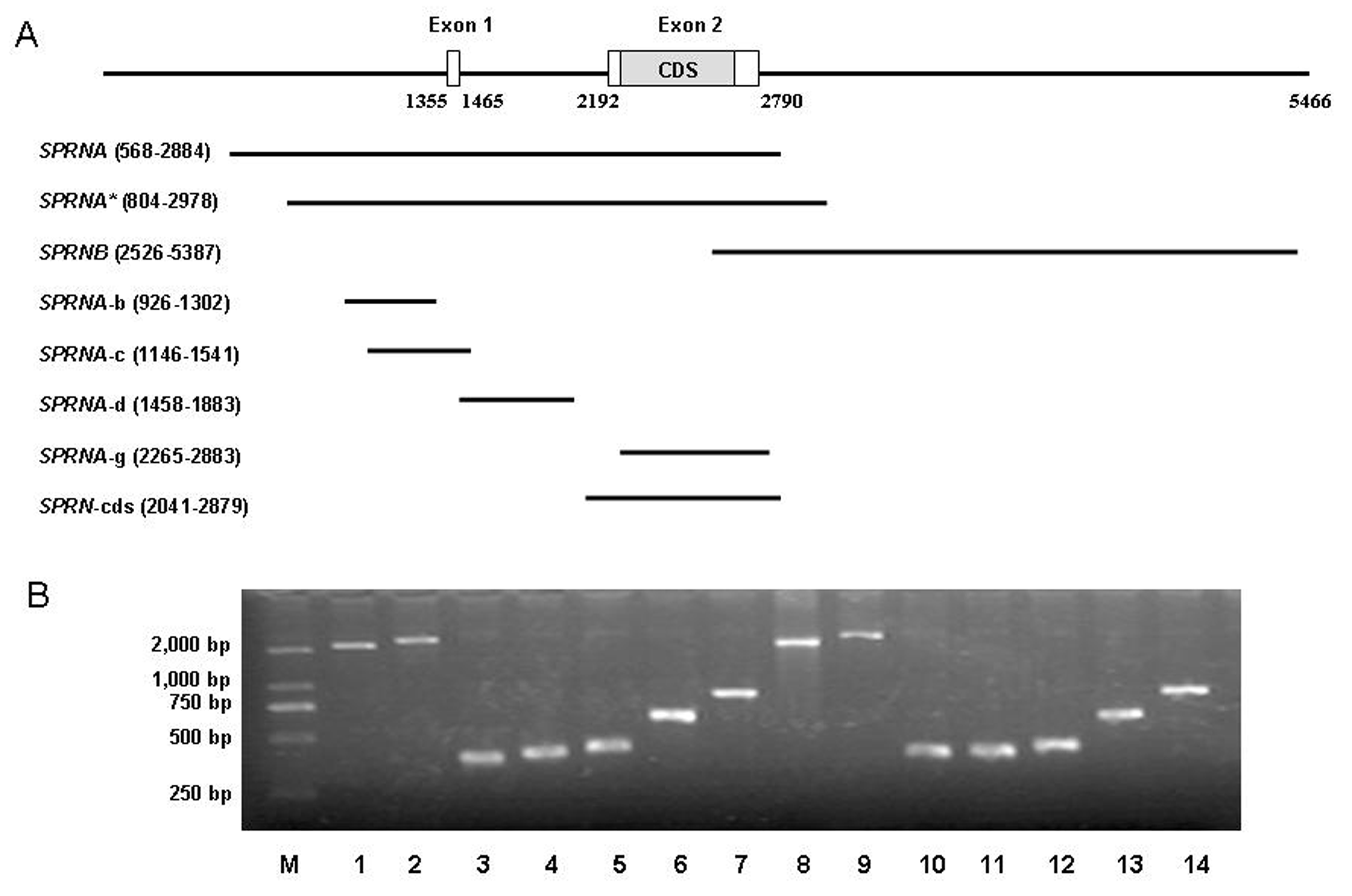

Supplement: Figure S1 — Detection of several amplicons of SPRN gene in cattle and buffalo. (A) Schematic representation of the PCR products of the bovine SPRN gene. The two exons of the bovine SPRN gene are represented with boxes. The coding sequence (CDS) region of SPRN (grey) is located on exon 2. * Indicates the buffalo-specific fragment amplified by PCR. (B) DNA samples from cattle (Lanes 1–7) and buffalo (Lanes 8–14) were amplified by PCR using the GC-RICH PCR System kit. The extracting PCR products of SPRNA (Lanes 1 and 8), SPRNB (Lanes 2 and 9) and SPRN-cds (Lanes 7 and 14) were directly sequenced. The high GC-rich fragments of SPRNA-b (Lanes 3 and 10), SPRNA-c (Lanes 4 and 11), SPRNA-d (Lanes 5 and 12) and SPRNA-g (Lanes 6 and 13) were cloned into the PMD 18-T vector and then sequenced. M: Molecular weight marker. (TIF) [file pone.0046601.s001.tif]

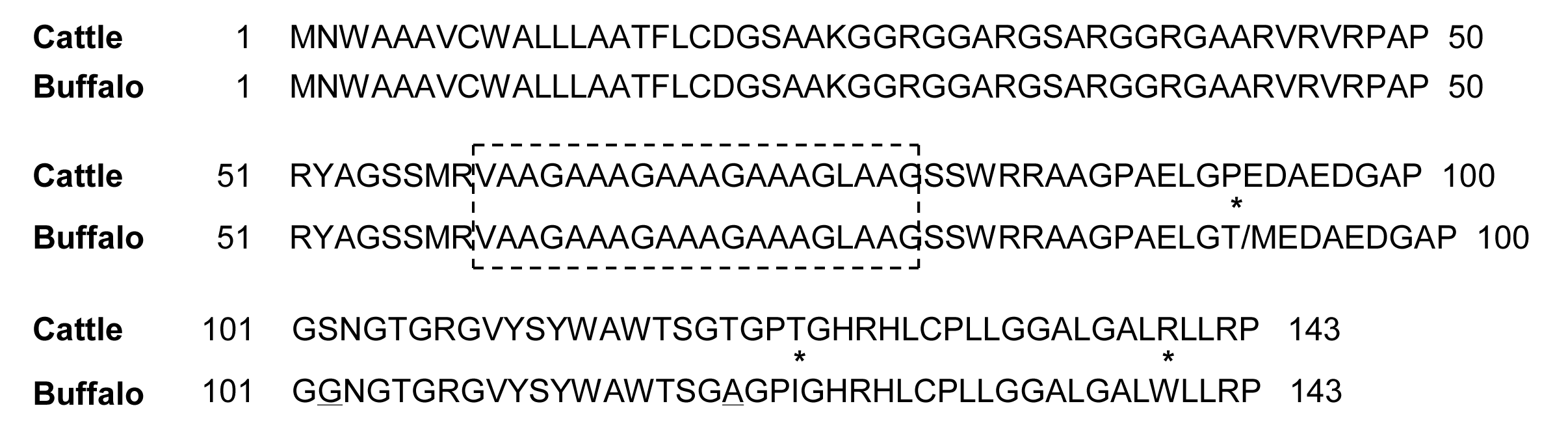

Supplement: Figure S2 — The predicted amino acid sequences of Shadoo in cattle and buffalo. The amino acid sequence of cattle Sho was translated from the SPRN gene sequence DQ058606 of GenBank. The amino acid sequence of buffalo Sho was produced according to the results of population analysis showing fixed differences (underlined) and significant differences (*) between the two species. Dashed box frame denotes the hydrophobic domain of Sho. (TIF) [file pone.0046601.s002.tif]
