# Supplementary material for: Comparative Analysis of the Shadoo Gene between Cattle and Buffalo Reveals Significant Differences
Source: PLoS One. 2012 Oct 10;7(10):e46601. doi: 10.1371/journal.pone.0046601 (PMC3468620; doi:10.1371/journal.pone.0046601)
Supplement: Table S1 — Amplicon characteristics of the PCR primers used in this study. (PDF) [file pone.0046601.s003.pdf]

Table S1. Amplicon characteristics of the PCR primers used in this study.

Primers	Forward primer (5'→3')<br>Reverse primer (5'→3')	Position #	Ta (℃)	Length (bp)	Mg^{2+} concentration (mM)	GC%
*SPRNA**	AGGGACCACAGGATCGAACAGACG<br>GGGACTAACCCCAGCAGGAGGCA	568-2884	55	2317	3.0	73.03
SPRNB	AGAGGCGTCTACAGCTACTGGGCGTGG<br>CCCCGTGATGCTGGACCGAGACC	2526-5387	60	2862	2.5	66.56
SPRNA †	GGAGCAGTCGGGTCACAGATGGT<br>AGCTGGGGTGGCCTGAACAGCCT	804-2978	55	2172	3.0	73.70
SPRNA-b	AGGGCTGGTGCTGCTCTG<br>ACGGGA GGGAGGGGACA	926-1302	54.3	377	3.0	70.03
SPRNA-c	GGGTGCGAGGTGCTGAAG<br>GACCAGAGGCGACCCCTG	1146-1541	54.3	396	2.5	77.78
SPRNA-d	CGCGGCTGGTAAGACCGG<br>TGTGGAGCGTGGAGTGCG	1458-1883	54.3	426	3.0	77.00
SPRNA-e	CTCACCCGCTTCCTTTGG<br>GACGGGGCACTCAGGAGCT	1711-2155	54.3	446	2.5	70.85
SPRNA-g	GACGGCAGTGCAGCCAAG<br>GGACTAACCCCAGCAGGAGG	2265-2883	55.4	619	3.0	76.58
SPRN-cds	CCCGCG TACCACCCAATG<br>TAACCCCAGCAGGAGGCA	2041-2879	56	839	2.5	73.90
Promoter 1*	CTCGAGCAGTCGGGTCACAGATGGTGGG<br>AAGCTTGCAGGGAGC CTCGGTCGC TTTC	808-1691	55	884	2.5	75.00
Promoter 1†	CTCGAGCAGTCGGGTCACAGATGGTGGG<br>AAGCTTGCAGGGAGCCTCGGTCGCTCTC	808-1691	60	883	2.5	74.63
Promoter 2	CTCGAGGAGCAGTCGGGTCACAGATGG<br>AAGCTTGAGGACTTGGGGAGGTAGGGAG	805-2129	58	1325	3.0	73.66

* and † indicate the PCR primers for cattle and buffalo, respectively. Other primers are universal for the two species.

# indicates position in sequence DQ058606 of GeneBank.
Ta = annealing temperature; GC% = the content of guanine plus cytosine.
