# Supplementary material for: Comparative Analysis of the Shadoo Gene between Cattle and Buffalo Reveals Significant Differences
Source: PLoS One. 2012 Oct 10;7(10):e46601. doi: 10.1371/journal.pone.0046601 (PMC3468620; doi:10.1371/journal.pone.0046601)
Supplement: Table S2 — Primers used for sequencing SPRN gene in cattle and buffalo. (PDF) [file pone.0046601.s004.pdf]

Table S2. Primers used for sequencing *SPRN* gene in cattle and buffalo.

**Primer**	**Sequence(5'→3')**
SPRNA_568-F *	AGGGACCACAGGATCGAACAGACG
SPRNA_804-F†	GGAGCAGTCGGGTCACAGATGGT
SPRNA_926-F	AGGGCTGGTGCTGCTCTG
SPRNA_1301-R	ACGGGAGGGAGGGGACA
SPRNA_1146-F	GGGTGCGAGGTGCTGAAG
SPRNA_1541-R	GACCAGAGGCGACCCCTG
SPRNA_1458-F	CGCGGCTGGTAAGACCGG
SPRNA_1883-R	TGTGGAGCGTGGAGTGCG
SPRNA_1711-F	CTCACCCGCTTCCTTTGG
SPRNA_2155-R	GACGGGGCACTCAGGAGCT
SPRNA_2009-F	AGCCCACTCCCCACTCCT
SPRNA_2503-R	GCTCCGTCTTCTGCATCCTC
SPRNA_2265-F	GACGGCAGTGCAGCCGAAG
SPRNA_2883-R *	GGGACTAACCCCAGCAGGAGGCA
SPRNB_2526-F	AGAGGCGTCTACAGCTACTGGGCGTGG
SPRNA_2978-R†	AGCTGGGGTGGCCTGAACAGCCT
SPRNB_3236-R	GGTGAGGGTGTTCTGGGAGT
SPRNB_3146-F	TCACCCATCCAGCAAGAAG
SPRNB_3703-R	GATAATTTGAGACCCTGACTG
SPRNB_3679-F	GTTGCAGTCAGGGTCTCAAATT
SPRNB_3978-R	GCGTCTGAGGCTGAGCACA
SPRNB_4232-F	GGGCGCAGCAGGACCTAGA
SPRNB_4472-R	GCGAGCACCTTCCAGCTAAT
SPRNB_4387-F	GTGCAGGCTGTGGTGGTG
SPRNB_5074-R	GCTGGCATTTTCTCAGGAGTT
T-vector-F	CGCCAGGGTTTTCCCAGTCACGAC
T-vector-R	AACAGGC TATGACCATG
RVprimer3	CTAGCAAAATAGGCTGTCCC
GLprimer2	CTTTATGTTTTTGGCGTCTTCCA

* and † indicate the primers used for sequencing the cattle and buffalo gene, respectively.
