# Supplementary material for: Comparative Analysis of the Shadoo Gene between Cattle and Buffalo Reveals Significant Differences
Source: PLoS One. 2012 Oct 10;7(10):e46601. doi: 10.1371/journal.pone.0046601 (PMC3468620; doi:10.1371/journal.pone.0046601)
Supplement: Table S3 — Overview of the fixed differences in the 3′ region of SPRN gene between cattle and buffalo. (PDF) [file pone.0046601.s005.pdf]

Table S3. Overview of the fixed differences in the 3’ region of *SPRN* gene between cattle and buffalo.

**Position#**	**Cattle**	**Buffalo**	**Position**	**Cattle**	**Buffalo**
g.2654	A	G	g.3881	C	T
g.2703	G	del	g.3882	A	G
g.2710	T	C	g.3884	C	G
g.2780	A	G	g.3995	C	T
g.2782	G	C	g.4019	G	A
g.2798	T	C	g.4035	G	C
g.2800	G	C	g.4044	C	T
g.2850	C	T	g.4047	C	T
g.2895	A	G	g.4050	T	A
g.2904	G	C	g.4056	G	T
g.2921	A	G	g.4059	G	A
g.2923	C	T	g.4070	C	A
g.3014	A	G	g.4081	C	T
g.3028	C	T	g.4084	C	T
g.3029	T	G	g.4098	G	A
g.3130	C	T	g.4108	A	C
g.3158	C	A	g.4109	C	A
g.3242	C	T	g.4110	C	T
g.3278	del	G	g.4115	C	T
g.3299	T	C	g.4162	G	A
g.3336	A	G	g.4232	G	A
g.3350	C	T	g.4233-44235	GGC	del
g.3359	T	G	g.4275	C	T
g.3390	A	G	g.4344	C	T
g.3421	del	CTTTT	g.4350	G	T
g.3449	C	T	g.4356	G	C
g.3504	A	G	g.4430	C	A
g.3525	G	A	g.4437	C	T
g.3540	C	T	g.4444	C	T
g.3547	A	G	g.4480	A	G
g.3596	G	A	g.4506	C	A
g.3601	T	C	g.4751	G	A
g.3665	A	C	g.4789	T	C
g.3750	A	G	g,4818	A	G
g.3758	C	A	g.4974	A	G
g.3764	C	G	g.4994	G	A
g.3832	A	G	g.5010	T	C
g.3876	A	G			

Position #: indicates position in sequence DQ058606 of GenBank.
