# Supplementary material for: Comparative Analysis of the Shadoo Gene between Cattle and Buffalo Reveals Significant Differences
Source: PLoS One. 2012 Oct 10;7(10):e46601. doi: 10.1371/journal.pone.0046601 (PMC3468620; doi:10.1371/journal.pone.0046601)
Supplement: Table S4 — Distributions of genotype frequency of polymorphisms in cattle and buffalo breeds. (PDF) [file pone.0046601.s006.pdf]

Table S4. Distributions of genotype frequency of polymorphisms in cattle and buffalo breeds.

		Cattle			Buffalo			
**Mutation**	**Location**	**Homo 1**	**Hetero**	**Homo 2**	**Homo 1**	**Hetero**	**Homoe 2**	**Amino acid change**
g.1018G>A	5’ region	0.765	0.147	0.088	1.000	0.000	0.000	
g.1077G>C		1.000	0.000	0.000	0.500	0.227	0.273	
g.1079C>A		1.000	0.000	0.000	0.545	0.091	0.364	
g.1111C>A		0.971	0.029	0.000	1.000	0.000	0.000	
g.1164C>A		0.441	0.206	0.353	0.000	0.000	1.000	
g.1244C>A		1.000	0.000	0.000	0.545	0.000	0.455	
g.1245T>C		1.000	0.000	0.000	0.773	0.045	0.182	
g.1304C>T		1.000	0.000	0.000	0.955	0.045	0.000	
g.1329G>A		1.000	0.000	0.000	0.955	0.045	0.000	
g.1404C>T	Exon 1	0.971	0.029	0.000	1.000	0.000	0.000	
g.1426T>C		0.971	0.029	0.000	1.000	0.000	0.000	
g.1483insA	Intron 1	0.000	0.000	1.000	0.000	0.136	0.864	
g.1488insG		0.000	0.000	1.000	0.000	0.136	0.364	
insGGG		-	-	-	0.000	0.091	0.364	
insGGGG		-	-	-	0.000	0.045	0.000	
insGGGGG		-	-	-	0.000	0.091	0.000	
insGGGGGG		-	-	-	0.000	0.182	0.000	
insGGGGGGG		-	-	-	0.000	0.045	0.000	
insGGGGGGAGGGG		-	-	-	0.000	0.045	0.000	
g.1544C>T/del		1.000	0.000	0.000	0.000	0.045/0.045	0.545/0.364	
g.1588A>G		1.000	0.000	0.000	0.864	0.045	0.091	
g.1599G>T		0.971	0.029	0.000	1.000	0.000	0.000	
g.1604A>G		1.000	0.000	0.000	0.864	0.045	0.091	
g.1614A>T		0.794	0.088	0.118	1.000	0.000	0.000	
g.1629C>A		1.000	0.000	0.000	0.909	0.000	0.091	
g.1660A>G		1.000	0.000	0.000	0.955	0.045	0.000	
g.1664Gdel		1.000	0.000	0.000	0.318	0.045	0.636	
g.1703C>A		0.676	0.206	0.118	1.000	0.000	0.000	
g.1818G>A		1.000	0.000	0.000	0.955	0.045	0.000	
g.1847G>A		1.000	0.000	0.000	0.500	0.136	0.364	

g.1940C>T		1.000	0.000	0.000	0.455	0.182	0.364	
g.1949C>T		0.912	0.088	0.000	1.000	0.000	0.000	
g.1997C>T		1.000	0.000	0.000	0.818	0.091	0.091	
g.2077G>A		1.000	0.000	0.000	0.455	0.091	0.455	
g.2303T>C	CDS	1.000	0.000	0.000	0.682	0.136	0.182	
g.2408C>G		0.941	0.059	0.000	1.000	0.000	0.000	
g.2418insCGCGGCGGGGGC		0.971	0.029	0.000	1.000	0.000	0.000	70insAAAG
g.2495A>G		0.324	0.088	0.588	1.000	0.000	0.000	
g.2564G>A		1.000	0.000	0.000	0.545	0.364	0.091	
g.2572C>T		0.971	0.029	0.000	0.455	0.182	0.364	Thr122Ile
g.2622C>T		1.000	0.000	0.000	0.955	0.045	0.000	Arg139Trp
g.2653C>T	3' region	1.000	0.000	0.000	0.500	0.136	0.364	
g.2655T>C		1.000	0.000	0.000	0.500	0.136	0.364	
g.2697C>A		1.000	0.000	0.000	0.818	0.182	0.000	
g.2711C>T		1.000	0.000	0.000	0.955	0.045	0.000	
g.2759C>A/T		1.000	0.000	0.000	0.909	0.045/0.045	0.000	
g.2826C>T		1.000	0.000	0.000	0.682	0.136	0.182	
g.2827C>T		1.000	0.000	0.000	0.955	0.045	0.000	
g.2865C>T		0.971	0.029	0.000	1.000	0.000	0.000	
g.2880G>A		1.000	0.000	0.000	0.409	0.136	0.455	
g.2914T>G		1.000	0.000	0.000	0.727	0.091	0.182	
g.2959C>T		0.882	0.059	0.059	1.000	0.000	0.000	
g.2966G>C		0.941	0.059	0.000	1.000	0.000	0.000	
g.2978T>A		1.000	0.000	0.000	0.682	0.136	0.182	
g.2979C>T		1.000	0.000	0.000	0.682	0.136	0.182	
g.2995G>C		1.000	0.000	0.000	0.682	0.136	0.182	
g.3045C>T		1.000	0.000	0.000	0.818	0.091	0.091	
g.3121C>T		0.324	0.147	0.529	0.000	0.000	1.000	
g.3165G>A		0.647	0.059	0.294	1.000	0.000	0.000	
g.3248C>G		1.000	0.000	0.000	0.136	0.045	0.818	
g.3251A>G		1.000	0.000	0.000	0.864	0.045	0.091	
g.3256T>C		1.000	0.000	0.000	0.136	0.045	0.818	
g.3281C>T		1.000	0.000	0.000	0.136	0.045	0.818	
g.3282A>G		1.000	0.000	0.000	0.136	0.045	0.818	

g.3301C>G	1.000	0.000	0.000	0.136	0.045	0.818
g.3304T>C	0.971	0.029	0.000	0.000	0.000	1.000
g.3327A>G	0.971	0.029	0.000	0.182	0.091	0.727
g.3353G>A	0.647	0.176	0.176	1.000	0.000	0.000
g.3381G>A	1.000	0.000	0.000	0.864	0.045	0.091
g.3421insG	0.000	0.000	1.000	0.864	0.045	0.091
g.3445C>G	1.000	0.000	0.000	0.182	0.091	0.727
g.3446C>G	0.971	0.029	0.000	0.000	0.000	1.000
g.3482G>A	0.971	0.029	0.000	1.000	0.000	0.000
g.3541G>A	0.912	0.088	0.000	1.000	0.000	0.000
g.3577C>T	0.971	0.029	0.000	1.000	0.000	0.000
g.3579T>C	0.353	0.176	0.471	1.000	0.000	0.000
g.3605T>C	0.971	0.029	0.000	1.000	0.000	0.000
g.3618T>C	1.000	0.000	0.000	0.955	0.045	0.000
g.3745G>A	1.000	0.000	0.000	0.227	0.136	0.636
g.3758C>A	0.971	0.029	0.000	1.000	0.000	0.000
g.3796A>G	1.000	0.000	0.000	0.955	0.045	0.000
g.3824C>T	1.000	0.000	0.000	0.909	0.091	0.000
g.3825G>A	0.941	0.059	0.000	1.000	0.000	0.000
g.3955C>T	0.794	0.147	0.059	1.000	0.000	0.000
g.3956C>A	1.000	0.000	0.000	0.818	0.091	0.091
g.3995C>T	0.971	0.029	0.000	0.000	0.000	1.000
g.4029C>T	1.000	0.000	0.000	0.909	0.000	0.091
g.4053T>C	0.882	0.059	0.059	1.000	0.000	0.000
g.4056G>C	0.824	0.059	0.118	1.000	0.000	0.000
g.4065C>T	0.735	0.088	0.176	1.000	0.000	0.000
g.4074C>A	1.000	0.000	0.000	0.636	0.091	0.273
g.4097G>A	0.794	0.088	0.118	1.000	0.000	0.000
g.4103A>G	1.000	0.000	0.000	0.727	0.000	0.273
g.4169C>T	0.618	0.147	0.235	1.000	0.000	0.000
g.4180C>T	1.000	0.000	0.000	0.818	0.091	0.091
g.4233G>A/T	0.824	0.029/0.088	0/0.059	-	-	-
g.4289G>A	0.941	0.059	0.000	1.000	0.000	0.000
g.4319G>C	0.971	0.029	0.000	1.000	0.000	0.000

g.4349A>G	1.000	0.000	0.000	0.818	0.091	0.091
g.4356G>C	0.971	0.029	0.000	1.000	0.000	0.000
g.4472C>G	1.000	0.000	0.000	0.182	0.000	0.818
g.4499A>T	0.941	0.059	0.000	1.000	0.000	0.000
g.4542C>A/T	1.000	0.000	0.000	0.818	0.091/0.091	0.000
g.4567G>A	0.794	0.088	0.118	1.000	0.000	0.000
g.4585G>A	1.000	0.000	0.000	0.818	0.091	0.091
g.4618-4619del	0.706	0.294	0.000	0.000	0.000	1.000
g.4721G>A	1.000	0.000	0.000	0.455	0.273	0.273
g.4763A>C	0.971	0.029	0.000	1.000	0.000	0.000
g,4780C>T	0.971	0.029	0.000	1.000	0.000	0.000
g.4810A>G	1.000	0.000	0.000	1.000	0.000	0.000
g.4819C>T	1.000	0.000	0.000	0.773	0.227	0.000
g.4838T>G	1.000	0.000	0.000	0.545	0.273	0.182
g.4902C>T	0.971	0.029	0.000	1.000	0.000	0.000
g.4903G>A	1.000	0.000	0.000	0.409	0.318	0.273
g.4950G>A	1.000	0.000	0.000	0.455	0.182	0.364
g.4964G>A	1.000	0.000	0.000	0.136	0.227	0.182
g.4992C>T	1.000	0.000	0.000	0.636	0.182	0.182
g.5001G>T	1.000	0.000	0.000	0.409	0.136	0.455

Homo = Homozygote; Hetero = Heterozygous**;** ins = insertion; del = deletion.
